# Foregut microbiology of the Arabian camel (*Camelus dromedarius*)

**DOI:** 10.1093/af/vfac049

**Published:** 2022-08-12

**Authors:** Rafat Al Jassim

**Affiliations:** Centre for Animal Science, Queensland Alliance for Agriculture and Food Innovation (QAAFI), The University of Queensland, St Lucia 4072, Brisbane, Australia

**Keywords:** Arabian camel, bacterial community, *Camelus dromedarius*, co-grazing, protozoa

**Implications**

- Our studies using the culture-dependent techniques provided vital information on the identity of important culturable bacterial species and their biochemical and physiological characteristics.
- Our studies revealed the novelty of the bacterial community of the camel’s forestomach. Most of the sequences generated are related to unknown bacterial species and do not match any sequences in the public database.
- Co-grazing camels with cattle has altered the forestomach bacterial community in camels and cattle.
- The results of our co-grazing study suggest further investigations into the transfer of forestomach microorganisms between the two species and the impact of bacterial transfer on digestion in cattle.

## Introduction

The Arabian camel (*Camelus dromedarius*) evolved as a foregut fermenter herbivore with the capacity to utilize fiber-rich diets. As a browsing animal, the camel spends time foraging and selects its own diet from available shrubs and trees that are often rich in antinutritional secondary compounds, which makes them unpalatable for many other species of animals. This feeding behavior reduces the competition for feed resources with other domesticated animals, particularly ruminant animals and horses.

It is important to emphasize that the Arabian camel belongs to the family Camelidae in the sub-order Tylopoda (Latin: padded foot) and the order Artiodactyla, which is distinct from the sub-order Ruminantia (Fowler, 2008). However, the two related evolutionary lineages share similarities and bear differences due to the parallel evolution they have undergone.

The evolution of camelids is dated back to the Eocene epoch, about 40 to 50 million yr ago (Fowler, 2008; Burger, 2016), and the divergence of Tylopoda and Ruminantia occurred early in the evolution process. The first ancestors of both groups were found in North America, as small goat-sized animals with simple stomachs (Fowler, 2008). The development of the compartmental stomach as a storage and fermentation vat occurred during the Miocene epoch.

Gut microbiota of the foregut fermenters, such as the camel and the cow, are involved in the digestion and synthesis of essential nutrients, detoxify antinutritional plant secondary compounds, and contribute to health. Knowledge of the microbial composition and function will assist in the management of the host animals to achieve high productivity while maintaining the health and well-being of the animal. Results of our research have established the identity of important bacterial groups, including lactic acid-producing (**LAB**) and lactic acid-utilizing bacteria (**LUB**), and cellulolytic bacteria. The different types of protozoa were also determined by microscopic examination. Bacterial diversity was investigated by constructing clone libraries and later by new-generation sequencing technologies and genome analysis.

The focus of this article is on the microbial community of the forestomach of the Arabian camel (*Camelus dromedarius*) to report the results of our research programs over the past two decades and to shed some light on the development in the field.

## The Compartmental Stomach of the Camel

**Figure 1.**
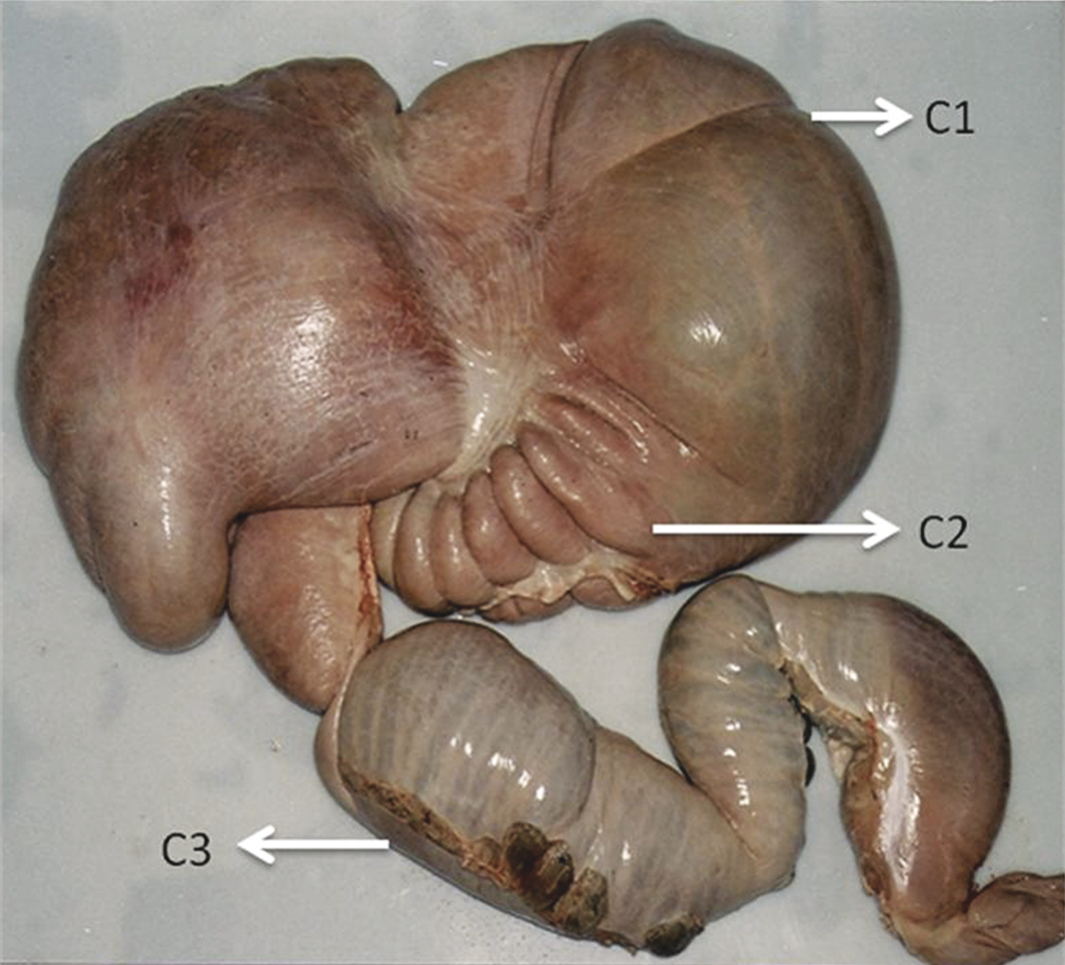
The compartmental stomach of the Arabian camel (*Camelus dromedarius*). Compartment 1 (C1), compartment 2 (C2), and compartment 3 (C3).

The compartmental stomach of the camel (Figure 1) consists of three compartments: C1, the voluminous part (~80% of the forestomach volume); C2, which resembles the reticulum in the ruminant stomach; and C3, which is a long tubiform with the distal part being the only hydrochloric acid secretory region (Engelhardt et al., 1988; Lechner-Doll et al., 1995). The omasum is missing from the camel’s forestomach, and all compartments have glandular epithelium. The internal lining of the compartmental stomach is smooth and not papillated. A large proportion of the forestomach epithelium consists of columnar surface epithelium and deep tubular glands (Lechner-Doll et al., 1995). The motility of the forestomach in camels is different from that in cattle but, in both species, it leads to the mixing of digesta and facilitates the passage of digesta into C3 and the intestines.

## The Microbial Ecology of the Forestomach of the Arabian Camel

The camel’s forestomach is home to a vast number and diverse microbiota, including bacteria, protozoa, fungi, and archaea. The relationship between the host camel and microbiota that inhabit the forestomach is a symbiotic relationship. The camel provides the physiologic conditions (temperature, pH, supply of substrates, and mixing) optimal for the survival and growth of the microbiota, while the microbiota break down the structural components of feed, detoxify certain compounds, synthesize essential nutrients, and contribute to health. As reported earlier (Al Jassim and Hogan, 2013), studies into the microbial communities of the forestomach of the Arabian camel were carried out at the Gut Microbiology Laboratory, University of Queensland, during the years 2003–2016. Early work aimed at identifying and characterizing bacteria involved in the development of fermentative acidosis in the foregut of camels and identifying lactate utilizers for use in feedlot cattle to reduce or prevent the risk of acidosis. The culturing of bacteria was carried out using culture-dependent techniques in roll tubes, while identification was carried out using molecular techniques based on the 16S rRNA gene (Ghali et al., 2004, 2011). Moving on with the technology, further studies applied culture-independent molecular techniques, first by constructing clone libraries and later by using new generation sequencing technologies and genome analysis (Samsudin et al., 2011, 2012, 2014; Suyub, 2014, Seshadri et al., 2018; Rabee et al., 2020).

Despite all that effort, we still believe that more studies are required, and the microbial ecosystem of the camel is still poorly investigated.

## Lactic Acid-Producing and Lactic Acid-Utilizing Bacteria

Work in our laboratory identified *Streptococcus bovis* as the main LAB and established the similarity of the camel isolates of *S. bovis* to those isolated from cattle, deer, and sheep (Ghali et al., 2004). Other LAB include *Selenomonas ruminantium*, *Butyrivibrio fibrisolvens*, *Lachnospira pectinoschiza*, and *Prevotella ruminicola* (Table 1). Of particular interest are the bacteria that produce d-lactate and those that produce both isomers l- and d-lactate. Isolates identified as *Selenomonas ruminantium* seem to have a complex genetic machinery that enables them to produce l-lactate, then shift to d-lactate production, and when it runs out of carbohydrate substrate, it converts lactate to propionate (Ghali et al., 2011). Such biochemical characteristics may qualify these isolates for the development of probiotics to prevent or reduce the risk of acidosis. Among other isolates of particular interest is *B. fibrisolvens* which produces large quantities of butyric acid, and this may explain the higher level of butyric acid often found in the forestomach fluid of the camel. *Lactococcus garvieae* and *Clostridium ramosum* are both important l-lactate producers.

**Table 1.** Fermentation end products of glucose supplement incubated in vitro in a broth medium of BM10† with the predominant bacterial isolates and incubated anaerobically at 39 °C for 20 h

Isolate	Fermentation end products (main products in bold)
*Streptococcus bovis*	**l** **-Lactate**, acetic acid, propionic acid, butyric acid
*Selenomonas ruminantium*	**l** **-Lactate**, **d****-lactate**, **acetic acid**, **propionic acid**, butyric acid
*Butyrivibrio fibrisolvens*	**Acetic acid**, propionic acid, **butyric acid**
*Lachnospira pectinoschiza*	**d** **-Lactate**, **acetic acid**, propionic acid, butyric acid
*Prevotella ruminicola*	**Acetic acid**, propionic acid, butyric acid
*Lactococcus garvieae*	**l** **-Lactate**
*Clostridium ramosum*	**l** **-Lactate**, **acetic acid**, propionic acid, butyric acid
*Staphylococcus epidermidis*	**l** **-Lactate**, **acetic acid**, propionic acid, butyric acid
*Pseudobutyrivibrio ruminis*	l-Lactate, **acetic acid**, propionic acid, **butyric acid**

^†^BM10 = Basal medium 10 (Caldwell and Bryant, 1966).

Further analysis of the bacterial community using a clone library approach revealed that 97% of the constructed operational taxonomic units (**OTU**s) were novel and were not related to any known sequences in the public database.

Analysis at the phylum level showed that the majority of these OTUs (67%) were affiliated with the phylum Firmicutes, followed by Bacteroidetes (25%). The remaining phyla were represented by Actinobacteria, Chloroflexi, Cynophyta, Lentisphaerae, Planctomycetes, Proteobacteria, and Sphirochaetes (Samsudin et al., 2011). Moreover, 11 clones of the 267 clones cultivated were identified as *Brevundimonas* sp., *Butyrivibrio fibrisolvens*, *Prevotella* sp., and *Ruminococcus flavefaciens*.

The phylum Firmicutes was represented by 38 phylotypes with the class Bacteroides being dominant. Further analysis of the Firmicutes taxon revealed 12 families within this phylum, including Acidaminococcaceae, Bacillaceae, Catabacteriaceae, Clostridiaceae, Erysipelotrichaceae, Eubacteriaceae, Lachnospiraceae, Oscillospiraceae, Peptococcaceae, Peptostreptococcaceae, Spiroplasmataceae, and Turicibacteraceae. Further analysis at the genus level resulted in four main genera: *Oscillobacter*, *Clostridium*, *Ruminococcus*, and *Catabacter*.

Overall analysis of the constructed clone library produced 16 families. Four of them (i.e., Eubacteriaceae, Clostridiaceae, Prevotellaceae, and Lachnospiraceae) were dominant and represented 63% of the OTUs. Analysis of the bacterial community in the foregut of the dromedary camel using a 16S rRNA gene clone library revealed novel sequences that were not closely related to sequences previously deposited into the GenBank database.

## Fiber-Degrading Bacteria

Another investigation aimed at revealing the identity of fiber-degrading bacteria in the forestomach of the camel using culture-dependent techniques and the clone library approach. In this investigation, three types of fiber were used: filter paper (**FP**), cotton thread (**CT**), and neutral detergent fiber (**NDF**) as the only source of carbon enrichment in the broth medium. A total of 283 sequences were harvested and assigned to 33 OTUs at the ≥98% sequence identity criterion. Firmicutes was the dominant phylum in the CT- and FP-enriched medium, with 11 and 18 OTUs assigned for CT and FP, respectively. There were two OTUs in each assigned to the phylum Proteobacteria. Sequences from the NDF-enriched medium were assigned to 11 OTUs, with 8 of them affiliated with the phylum Proteobacteria. This clearly demonstrated the influence of fiber type in determining the bacterial species associated with different diets. In that investigation, a very low number of clones of *Fibrobacter succinogenes* were detected in the FP enrichment medium, and no cellulolytic ruminococci were detected in the three media (Samsudin et al., 2012). While some bacterial species were unique to fiber type, others were shared by the different fiber types. Interestingly, the species *Clostridium bifermentans* was shared by the three media types.

The study reported new bacterial species and possibly new genera. Our finding emphasized the need for further studies into this poorly investigated microbial ecosystem.

Analysis at the species level revealed that *Pseudobutyrivibrio ruminis* was the most abundant in CT-enriched medium (38.5%), while *Eubacterium* sp. was abundant in FP medium (32%) and *Ruminobacter amylophilus* was abundant in the NDF medium (36%).

Quantification of the two fiber-degrading bacteria *Fibrobacter succinogenes* and *Ruminococcus flavefaciens* using real-time polymerase chain reaction (qPCR) confirmed the low representation obtained earlier using clone library analysis. The cell numbers of *F. succinogenes* and *R. flavefaciens* in the foregut content of the camel were 2.03 × 10^3^ and 9.83 × 10^3^ cells mL^−1^, respectively (Samsudin et al., 2014). Together, they are present in less than 0.01% of the foregut contents.

A similar approach was followed by Rabee et al. (2022) who incubated ground barley straw with camel forestomach fluid anaerobically for 72 h, and then investigated the diversity and the structure of bacteria attached to barley straw using Illumina Mi-Seq sequencing of the V4-V5 region of 16S rRNA genes. Results revealed that the phyla Firmicutes and Bacteroidetes were the most dominant among the attached bacteria. At the genera level, the RC9_gut_group, *Ruminococcus*, *Saccharofermentans*, *Butyrivibrio*, *Succiniclasticum*, *Selenomonas*, and *Streptococcus* were dominant.

More recently, a report by Rabee (2022) showed that roughage type affects both the composition of the bacterial community and enzyme activity. The two phyla Bacteroidetes and Firmicutes were the dominant among bacteria. On the one hand, an Egyptian clover hay diet increased the proportions of the genera *Prevotella* and *Ruminococcus* and showed higher xylanase activity. On the other hand, a barley straw diet increased the genera *Butyrivibrio*, RC9_gut_group, and *Fibrobacteres* and produced greater cellulase activity.

Earlier studies in our laboratory using high-throughput pyrosequencing of 16S rRNA provided us with detailed information on the microbiome diversity and complexity of Australian feral camels (*Camelus dromedarius*). Results of the taxonomic analyses yielded 21 bacterial phylotypes with Bacteroidetes and Firmicutes being the most abundant with 56.5% and 19.3%, respectively (Suyub, 2014). Similarly, Bhatt et al. (2013) reported a relative abundance rate of 55.5 for Bacteroidetes in handfed camels. The similar estimates for the relative abundance of Bacteroidetes by these two studies, despite the difference in the feeding management of the camels (browsing on native vegetation vs handfeeding), suggest a relatively stable bacterial composition of the camel’s forestomach. The phylum Proteobacteria was the third most abundant group with a relative abundance of 6.7%. Other phyla including Spirochaetes, Lentisphaerae, and Fibrobacteres were also present, but at a much lower abundance.

Rabee et al. (2020), using total rRNA sequencing, have identified 12 phyla and showed that Firmicutes is the dominant phylum in the forestomach of Arabian camels. Bacterial communities associated with both the solid and liquid fractions of the forestomach digesta from camels on different diets were analyzed. Other phyla, including Bacteroidetes, Proteobacteria, Spirochaetes, and Fibrobacteres, were among the 5 most predominant phyla of the 12 phyla that were identified in their work. The relative abundance of microbes varied between liquid and solid fractions and was influenced by diet. Within the phylum Firmicutes, the two families Lachnospiraceae and Ruminococcuceae were the predominant. At the genus level, six genera dominated the phylum Firmicutes, including *Butyrivibrio*, RFN8-YE57, *Ruminococcus*, vadinHA42, *Acetitomaculum*, and *Blautia*.

Another investigation aimed at generating a microbial profile for the forestomach of the Indian camel using 16S rRNA gene amplicon and shotgun metagenomics technology was carried out by Hinsu et al. (2021). In their study, they investigated the effect of diet, digesta fraction, and breed of camel on bacterial diversity and function. A significant difference in alpha diversity (amplicon sequence variant and Shannon index) was observed between fractions, while the diet and the breed of camels had no effect on bacterial diversity. A total of 28 phyla were observed, and, as with previous investigations, Bacteroidetes and Firmicutes were the dominant phyla. Other phyla among the most abundant were Proteobacteria, Fibrobacterota, Firmicutes-C, and Verrucomicrobiota. For carbohydrate hydrolyzing enzymes, the glycoside hydrolases were the most abundant among all (Hinsu et al., 2021).

To identify potent cellulolytic and hemicellulolytic bacteria of camel forestomach origin, Srivastava et al. (2020) screened 6,716 bacterial cultures that were isolated from fresh (5,220) and enriched forestomach (1,496) fluids. The chromophoric substrate screening method enabled them to identify five isolates with the greatest endoglucanase activity. These isolates were identified as *Pseudomonas stutzeri*, *Paenibacillus dendritiformis*, *Citrobacter* sp., *Bacillus subtilis*, and *Enterobacter* sp.

**Figure 2.**
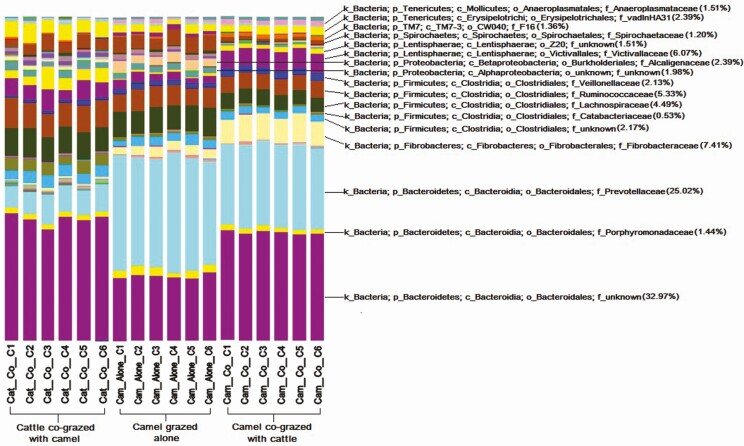
Bacterial distribution between cattle co-grazed with camels, camels grazed alone, and camels co-grazed with cattle evaluated at the family taxonomical level. Initial alphabet for each classification stands for P = phylum, C = class, O = order, and F = family, after Suyub (2014).

The effect of co-grazing camels with cattle on the bacterial community in both species has also been investigated (Suyub 2014). Co-grazing altered the community structure in both species of animals, with phylogenetic diversity differences between camels grazed alone and camels co-grazed with cattle and cattle co-grazed with camels were observed (Figure 2). Camels grazed alone showed the lowest species richness (Chao 1) when compared with the other two groups. The three groups of animals had similar community composition at the phyla level but different percentages of phylum composition. Sequences assigned to Bacteroidetes were the most abundant among the three groups, with camels co-grazed with cattle being the highest (60.5%). The lowest was for cattle co-grazed with camels (46.7%), while camels grazed alone had a relative abundance of 56.5%. For sequences assigned as Firmicutes, cattle co-grazed with camels had the highest rate of abundance at 24.8%, followed by 19.3% for camels grazed alone, and 14.8% for camels co-grazed with cattle. Significant differences were also observed between the three groups of animals in the abundance of the minor groups, Spirochaetes, Proteobacteria, Lentisphaerae, and Fibrobacteres. Fibrobacteres and Lentisphaerae were both higher in camels co-grazed with cattle, while Proteobacteria and Spirochaetes were higher in camels grazed alone. Differences were also observed at the family level.

The co-grazing study identified 433 OTUs that were only present in camels co-grazed with cattle and were showing 45.3 to 99.2% similarity to uncultured bacteria according to the ribosomal database project. This clearly demonstrated the influence of co-grazing on the microbial community of animal species involved and the potential impact of such changes on fermentation processes in the forestomach of these herbivores.

## Protozoa Population and Types in the Forestomach of the Arabian Camels

Early studies in our laboratory (Ghali, 2006), which were reported in a short communication (Ghali et al., 2019), gave an insight into the protozoan population in the forestomach of the Arabian camel (*Camelus dromedarius*) and the effect of grain supplementation and forestomach pH on the numbers and types of protozoa. Supplementary feeding of grain to a roughage diet decreased the forestomach pH, from an average of 6.4 to 5.3, and dramatically reduced the number of total protozoa. Under roughage feeding conditions, *Entodinium* spp. were the dominant species (86.3%), followed by *Eudiplodinium* spp. (7.3%) and Epidinium spp. (4.6%). Other species (i.e., *Dasytricha* spp., *Oligoisotricha* spp., and *Buetschlia* spp.) were represented by less than 2% of the total protozoa population. Grain feeding and the drop in forestomach pH have led to a dramatic decrease in the number of *Entodinium* spp. and an increase in *Epidinium* spp., while minor groups of protozoa were not detected when the forestomach pH dropped below 6.

In their comprehensive study using total rRNA sequence analysis, Rabee et al. (2020) reported two protozoan families in the forestomach of the camel: Ophryoscolecidae and Isotrichidae. Seven genera were classified under Ophryoscolecidae: *Diplodinium*, *Ophryoscolex*, *Entodinium*, *Polyplastron*, *Eudiplodinium*, *Epidinium,* and *Trichostomatia*, while two genera: *Dasytricha* and *Isotricha* were classified under Isotrichidae. There was a clear variation in the protozoal population among camels, but all camels had similar protozoal community composition with *Diplodinium, Ophryoscolex, and Entodinium* being the most dominant.

## Discussions

Results from our research programs have provided valuable information on the bacterial and protozoal community of the camel’s forestomach. Using the culture-dependent techniques enabled the harvest of important bacterial isolates representing the predominant LAB and LUB in the forestomach of the Arabian camel. The biochemical characteristics of these isolates were investigated and compared with similar isolates from other species of animals. The inclusion of eight camel bacterial isolates with the collection of rumen bacteria of the Hungate1000 project (Seshadri et al., 2018) was a significant achievement, obtaining detailed information not previously available on these isolates. While our studies have revealed the novel nature of the bacterial community of the camel’s forestomach, these bacteria play the same role as their counterparts found in the ruminants’ compartmental stomach. This is a clear outcome of the original ancestry and their parallel evolution. At higher taxonomic-level analyses, our studies showed that the two phyla Bacteroidetes and Firmicutes were the most abundant within the bacterial community. Despite the different feeding system (browsing vs. intensive feeding), our results agreed with those reported by Bhatt et al. (2013), who also reported the abundance of these two phyla. More work is required to link bacterial diversity with function and animal performance.

## Conclusion

Results from our studies revealed the novelty of the bacterial community of the camel’s forestomach and its diversity. While similar phyla numbers and types to those in cattle were observed, bacterial types at the OTUs levels (species level) were different. Mixed grazing with other species (i.e., camels co-grazed with cattle) has altered the bacterial community composition but maintained the same groups at the phyla level. Similar changes were observed at the family level. Protozoal types were like those in cattle, but further studies using independent molecular techniques may reveal a different story. More studies linking bacterial diversity and rumen function are needed.

## About the Author

**Dr Al Jassim** is an animal scientist with a specialization in Nutrition Biochemistry and Gut Microbiology who is holding an Honorary Associate Professor position at the Centre of Animal Science, Queensland Alliance for Agriculture and Food Innovation (QAAFI), The University of Queensland, Australia. During the past 25 years, the focus of his research programs was on the impact of commensal gut microorganisms in large animal health and disease and the role of gut microbial ecology in the nutrition of domesticated and wild herbivores. Interaction between diet and the microbial community of the intensively managed animals has been of particular interest. Recent research programs have dealt with a variety of topics including the use of plant extracts to control pathogenic microorganisms such as Campylobacter, isolation of bacteria with the ability to degrade mimosine and prevent Leucaena toxicity, monensin toxicity in camels, seasonal changes in tropical grass quality and selenium and vitamin E supplementation in camels.
